# Systematic Standardized and Individualized Assessment of Masticatory Cycles Using Electromagnetic 3D Articulography and Computer Scripts

**DOI:** 10.1155/2017/7134389

**Published:** 2017-09-18

**Authors:** Ramón Fuentes, Alain Arias, María Florencia Lezcano, Diego Saravia, Gisaku Kuramochi, Fernando José Dias

**Affiliations:** ^1^Department of Integral Dentistry, Research Centre in Dental Sciences (CICO), Dental School, Universidad de La Frontera, Temuco, Chile; ^2^Universidad Adventista de Chile, Chillán, Chile; ^3^Facultad de Ingenieria, Universidad Nacional de Entre Ríos, Entre Ríos, Argentina; ^4^Department of Restorative Dentistry, School of Dentistry, Universidad Finis Terrae, Santiago, Chile

## Abstract

Masticatory movements are studied for decades in odontology; a better understanding of them could improve dental treatments. The aim of this study was to describe an innovative, accurate, and systematic method of analyzing masticatory cycles, generating comparable quantitative data. The masticatory cycles of 5 volunteers (Class I, 19 ± 1.7 years) without articular or dental occlusion problems were evaluated using 3D electromagnetic articulography supported by MATLAB software. The method allows the trajectory morphology of the set of chewing cycles to be analyzed from different views and angles. It was also possible to individualize the trajectory of each cycle providing accurate quantitative data, such as number of cycles, cycle areas in frontal view, and the ratio between each cycle area and the frontal mandibular border movement area. There was a moderate negative correlation (−0.61) between the area and the number of cycles: the greater the cycle area, the smaller the number of repetitions. Finally it was possible to evaluate the area of the cycles through time, which did not reveal a standardized behavior. The proposed method provided reproducible, intelligible, and accurate quantitative and graphical data, suggesting that it is promising and may be applied in different clinical situations and treatments.

## 1. Introduction

The evolution of the human species, as expressed in increased body and brain size, is closely related to increased nutritional energy intake, achieved by improvements in the masticatory function and changes in food storage and preparation [1].

Chewing is one of the first and major steps in the digestive process of most mammals; it is characterized by a complex motor-sensory activity that consists of rhythmic opening and closing of the jaw to reduce, grind, and moisten the food, leading to the formation of a bolus that can be swallowed. Since this process is followed by digestion, it is an important factor for nutrition maintenance and feeding behavior [2–8]. This complex function integrates muscles, the temporomandibular joint (TMJ), tongue, palate, salivary glands, and especially periodontal and teeth, all of which are components of the stomatognathic system [9].

Rhythmic jaw movements are referred to as masticatory cycles or mandibular cycles, with each cycle consisting of two components: mouth opening and mouth closing. Each of these components can usually be subdivided into phases, for example, slow and fast opening, slow and fast closing [3]. The mastication sequence can be divided into three stages: early, middle, and late, in which chewing gradually changes [4]. Although mastication occurs bilaterally, many people present a preferred chewing side [10].

The functional significance of occlusal disturbances to the masticatory system has been investigated extensively, primarily because of the suggestion that occlusal disturbances might have a harmful effect on the masticatory system, for example, impairment of jaw function and balancing interferences; the latter presents a relatively high prevalence of approximately 16% in the adult dentate population [11]. Swallowing may also be related to changes in the masticatory system, especially if we consider the importance of lingual movements [12, 13].

The recording of mandibular movements was introduced into dentistry as a planning tool to analyze the movements' geometry in order to achieve the best results in treatment [14].

Mandibular movements occur in three dimensions; however, in the classic studies, they are described and analyzed in two dimensions [15]. In recent decades, technological improvements in position-tracking techniques have made it possible to record the dynamics of articulations with high temporal resolution [16]. An articulatory acquisition technique that allows more natural movement and offers high acquisition rates is electromagnetic articulography [17].

Electromagnetic articulography (EMA) devices are capable of measuring movements with fine spatial and temporal resolutions, providing useful articulatory data. The position of the receiver coil is detected in the EMA device on the basis of a field function representing the spatial pattern of the magnetic field in relation to the relative positions of the transmitter and receiver coils [18, 19].

In the past, methodological difficulties have restricted investigations involving the tracking and recording of articulatory movements. These difficulties, including the need to rely on techniques with ionizing radiation, have been largely overcome with advent of EMA, which represents a safe, noninvasive, and accurate method of tracking movements in human beings [16]. With the use and evolution of EMA, it has recently become possible to measure a large number of functions related to tongue-mandible movements in the three planes and in real time [15, 20].

Despite these advances, improvements, and developments in the analysis of masticatory cycles, the interpretation of these data is no simple task, since there is no software to carry out a simple, reproducible, and understandable analysis of the data generated by EMA or any other tracking method.

The aim of this study was to describe an innovative method for accurate, systematic analysis of masticatory cycles, using the 3D-EMA, and the multiparadigm numerical computing environment, MATLAB, for rapid, intuitive, and understandable analysis of the data obtained.

## 2. Materials and Methods

### 2.1. Electromagnetic Articulograph AG501 Description

**Figure 1:**
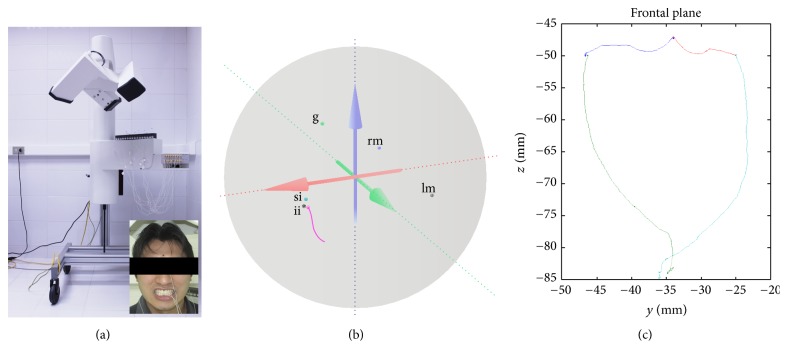
(a) AG501 3D Electromagnetic Articulograph (EMA) and properly positioned sensors: 2 mastoids (reference), 1 glabellar (reference), 1 upper incisor (reference), and 1 lower incisor (movement); (b) spherical volume of analysis (30 cm of diameter) showing the sensor positioned in volunteers (g: glabellar; rm: right mastoid; lm: left mastoid; si: superior incisor; ii^*∗*^: inferior incisor/movement) and an opening trajectory (pink line); (c) trajectory obtained from border mandibular movements in the frontal plane.

The Electromagnetic Articulograph (EMA) AG501 (Carstens Medizinelektronik, Lenglern, Germany) represents an evolution of two-dimensional (2D) predecessors (Carstens AG100 and AG200). It does not require a heavy restraining head mechanism and provides motion tracking with five degrees of freedom. 3D-EMA produces smaller measurement errors and allows a larger range of sensor positions and orientations than 2D-EMA, providing an unprecedented level of access to the most complex movements [16, 20–22]. The AG501 (Figure 1(a)) has three transmitter coils that detect magnetic fields to collect movement data in 3 dimensions at multiple points, enabling measurement in real time of the movements of the structures connected to the sensors within a 30 cm diameter spherical volume (Figure 1(b)) [15].

The AG501 EMA can be connected to a total of 16 sensors. According to the tests conducted to develop the protocols in this study, we defined the use of 5 sensors per individual. Once the measurements have been taken by the EMA, the data are extracted from the computer connected to the EMA and transferred to a second computer, where they are analyzed by MATLAB. The Electromagnetic Articulograph has been certified by Federal Communications Commission (independent US government agency) as a low-power communication device transmitter that uses electromagnetic fields with a frequency range of 7.5 to 13.75 KHz. This range is lower than the frequency range of radio transmission devices such as cellphones (10 MHz to 300 GHz) [23]. The local precision of AG501 was considered adequate for studies of speech articulatory movements [24], whose movements are finer compared to mandibular movements.

### 2.2. Calibration of AG501 EMA Device

To ensure correct performance of the AG501 EMA in recording movements, the sensors must be calibrated as indicated by the manufacturer. The calibration process is automatic and takes ~20 minutes. Calibration is performed once per sensor set. Once a sensor set is calibrated, it can be used in multiple recording sessions. Before every recording session, all the sensors must be coated with liquid silicone.

The sensors employed are classified by the operator as reference or movement sensors. The reference sensors identify the location/direction of the head in the three spatial planes. The movement sensors are those that will record the movements, with reproduction in the form of trajectory lines which will allow visualization of mandibular movements [15].

### 2.3. Preparation for Analysis

In this study, we evaluated 5 young adult volunteers, 3 women and 2 men (19 ± 1.7 years) with normooclusion (Angle Class I), dental college students. A questionnaire and clinical examination recommended by the “American Academy of Oralfacial Pain” (1993) were administered to the volunteers to certify the integrity of the temporomandibular joint. These volunteers were selected for their ease in understanding the mandibular movements and because they did not present any significant alteration in dental occlusion.

The experiment was conducted in the Oral Physiology Laboratory of the Research Centre with previous approval by the university Ethics Committee, following international law for human experimental procedures.

The movement recording session was initiated by performing a screening procedure, which consisted in the application of a questionnaire to collect information about possible problems with mandibular movement and problems associated with the oral cavity, as well as health status and oral care. A clinical evaluation was carried out subsequently to note the presence/absence of teeth, overbite and overjet values, and right and left molar and canine class and to evaluate the amplitude of mandibular movements and the presence of sensitivity or alterations to the TMJ or associated muscles.

Before the recording session, subjects were instructed and trained in the movements to be performed after fitting of the sensors. This training is important in this stage of the experiment, especially for subjects who have no knowledge of these movements.

Once the movement training was complete, the sensors were fitted to the subject with biologically compatible glue (Ethyl-2-Cyanoacrylate, Epiglu®, Meyer-Haake, Germany). The sensors were positioned at the following anatomical points (Figure 1(a)): (1) right mastoid process (reference sensor); (2) left mastoid process (reference sensor); (3) glabella (reference sensor); (4) upper incisor (between the two upper central incisors) (reference sensor); (5) lower incisor (between the two lower central incisors) (movement sensor). The coil system was lowered to 2 cm above the top of the subject's head. The subject was told to look forwards and adopt the postural head position, without slopes of the head, with a fixed stare to an anterior point, which reflected the more comfortable position to the patients, which was used to help standardize the registered mandibular movements.

The last step before starting the recordings was “Head Correction” to set the reference sensors (2 mastoids, 1 glabella, and 1 upper incisor). These sensors allow the system to eliminate head movements from the recordings and reflect the normalized position of the sensors placed on the mandible.

### 2.4. Protocols of Mandibular Movements

*Border Movements in the Frontal Plane*. In this protocol the subjects were instructed to perform the maximum right lateral contact movement; then, from this point, the subject performed the right maximum border aperture. Subsequently the subject repeated the same moves to the left side. The movements of the right and left side were performed starting from the maximum intercuspation position (MIP) and were repeated three times in different registers. The records of these movements allowed us to obtain the maximum displacement path in the frontal plane that would later serve as a comparison area for the whole masticatory cycle (Figure 1(c)).

*Masticatory Cycles*. The masticatory cycles were recorded by instructing subjects to chew a 3.5 g portion of peanuts. The movements were recorded from the start of chewing process (MIP) until the subject was ready for the first swallow; the number of cycles was not controlled and chewing happened naturally. Recording was repeated three times.

### 2.5. Data Analysis

Immediately after recording, the data were properly saved in the AG 501 EMA software, identified, and transferred from the recording computer to another computer for data analysis. To analyze the movements, custom MATLAB scripts were used. MATLAB (Matrix Laboratory) is an integrated development environment with its own programing language (M), widely known in the scientific field. It is used for machine learning, signal processing, image processing, computer vision, communications, control design, and other functions (MathWorks, Inc., USA). This software allows visualization of the movement trajectories recorded in all required planes.

*Analysis of the Morphology of Masticatory Cycles*. Masticatory cycles were analyzed using a specially prepared MATLAB script which separated the sets of cycle trajectories recorded during the act of chewing, making it possible to evaluate the morphology of each cycle individually, as well as the set of cycles as a whole.

*Quantitative Analysis*. The quantitative parameters of the cycles were evaluated as described follows:

- Area and number of cycles: the average area of each cycle and the number of masticatory cycles were obtained.
- Correlation between area and number of masticatory cycles: a correlation was made between the number of cycles and the average area of these cycles in each repetition of chewing peanuts for each subject.
- Ratio between the cycle area and the frontal mandibular border movements area (Ca/FMBMa): the ratio between the area of each individual cycle and the area of the figure formed by border mandibular movement from the front view was also obtained (frontal view of Posselt's envelope). For each subject only the figure that revealed the largest frontal mandibular border movements area (FMBMa) among the three repetitions was used for this ratio relating it to the cycle area (Ca).
- Distribution of areas of the cycles: finally, we observed the distribution area of the individual cycles during the chewing process.

## 3. Results

### 3.1. Morphology of Masticatory Cycles

**Figure 2:**
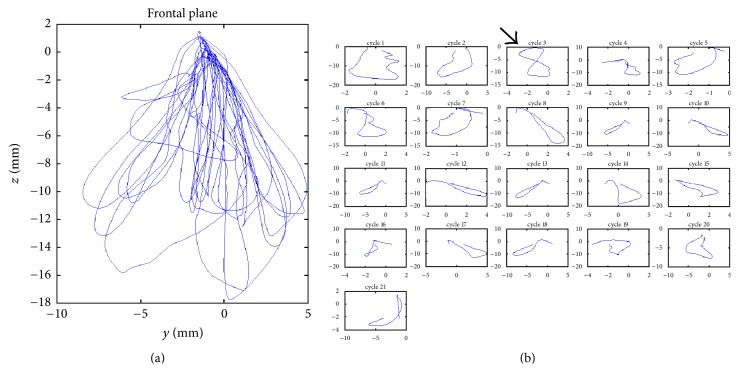
(a) Set of chewing cycle trajectories recorded during mastication. (b) Individualized trajectories of each chewing cycle.

The analysis of masticatory cycles provided images of the cycle trajectories set (Figure 2(a)), as well as showing the individual trajectories of each cycle (Figure 2(b)) in frontal view.

Morphological analysis of individual cycles revealed irregular cycles, sometimes forming a figure of 8 (Figure 2(b), cycle 3, arrow); however, the cycles tending to an elliptical shape were the most frequent, with varying amplitude, area, orientation, inclination, and prevalent side.

**Figure 3:**
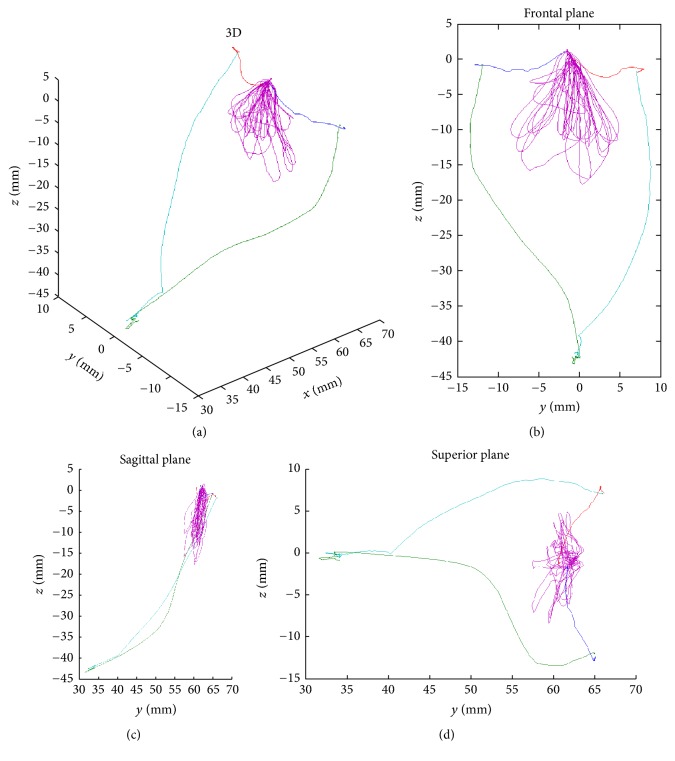
Analysis of set of cycles' trajectory associated with mandibular border movements in different views. (a) 3D analysis; (b) frontal plane view; (c) sagittal plane view; (d) superior plane view.

The set of cycles in three dimensions (Figure 3(a)), showing the front view (Figure 3(b)), sagittal view (Figure 3(c)), and horizontal view (Figure 3(d)) associated with the trajectory of mandibular border movements in the frontal plane (Posselt's frontal), were also obtained using this method.

A wide range of width and height was observed in the chewing cycles in which the main orientation was vertical or horizontal, and others tend to an inclination of 45°. The morphology of a number of cycles introduced MIP as the fulcrum of the movement, from which the subject moved the jaw to right and left.

### 3.2. Quantitative Analysis

The quantitative data obtained in this study are presented in Table 1.

### 3.3. Area and Number of the Cycles

The average number of cycles used by subjects to masticate 3.5 g peanuts ranged from 9 to 24. The areas of the masticatory cycles were obtained from the images formed by the trajectory of jaw movement in frontal plane (Figure 2(b)). The average of the areas of these trajectories ranged from 21.49 ± 19.11 to 74.67 ± 32.27 mm^2^ among 5 subjects in three repetitions.

### 3.4. Correlation between Area and Number of Masticatory Cycles

**Figure 4:**
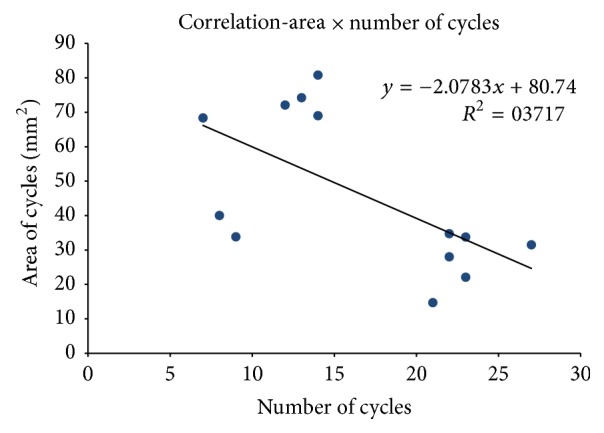
Scatter plot of correlation between the number and mean area of masticatory cycles. A negative correlation between the evaluated factors was noted, whose formula is shown in the chart.

The correlation between the number of cycles and the average area of each cycle in each repetition by subjects was calculated (Figure 4). The Pearson correlation index was −0.61, a moderate level of negative correlation [25] between the area and the number of the cycles showing that the greater the area, the smaller the number of masticatory cycles required for chewing peanuts.

### 3.5. Ratio between Cycle Area and Frontal Mandibular Border Movements Area (Ca/FMBMa)

The ratio Ca/FMBMa of each cycle was compared with the area of the frontal mandibular border movements (FMBMa); the average of these values with standard deviation is shown in Table 1 as a percentage. The values of the ratio varied among the subjects evaluated from 3.71 ± 3.38 to 19.08 ± 8.24%.

### 3.6. Distribution of Cycle Areas

**Figure 5:**
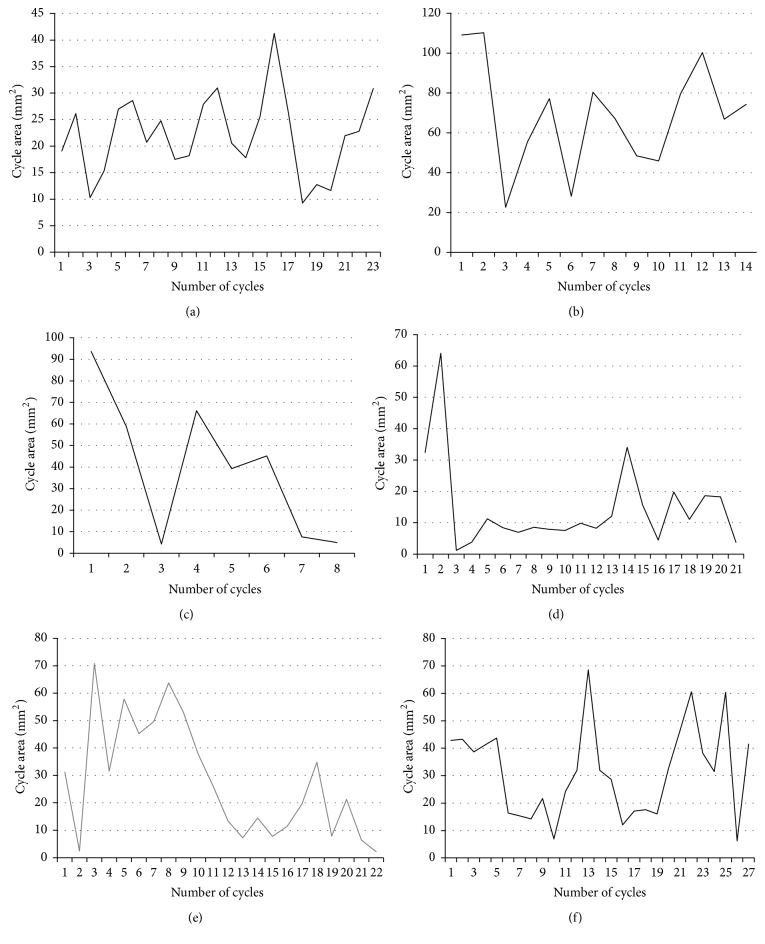
Distribution of the areas of individualized cycles. (a) Subject 1; (b) Subject 2; (c) Subject 3; (d) Subject 4, repetition 1; (e) Subject 4, repetition 2 (grey line); (f) Subject 5.

The distribution of the cycle areas during the chewing process was also analyzed in this study. It was observed that this distribution pattern varied between subjects, as was revealed by the range of variation of the areas. Subject 1 (Figure 5(a)) revealed maximum amplitude variation of approximately 30 mm^2^ (~10–40 mm^2^); Subject 2 (Figure 5(b)) showed variation which exceeded 90 mm^2^ (~20–110 mm^2^); Subject 3 (Figure 5(c)) also showed a wide variation in the area of cycles (~5–95 mm^2^) during the chewing process in a reduced number of cycles. Subject 4 (Figures 5(d) and 5(e)) revealed differences in the distribution pattern of these areas in different repetitions; however there was little variation in the number of cycles and their amplitude. Subject 5 (Figure 5(f)) showed a smaller variation range in the areas of the cycles (~5 to 70 mm^2^) than Subjects 2 and 3 and the largest number of cycles.

## 4. Discussion

The methodology proposed in this study used the AG 501 EMA and scripts especially developed in MATLAB to analyze the masticatory cycles of young subjects, Class I normoocclusion. The present method provided graphical data of the masticatory cycles trajectory and quantitative data simply, objectively, and accurately, allowing further detailed assessment of these functional movements [15, 22].

New applications for instrumental analysis of mandibular movements imply recording the functional movement capacity and the coordination of mandibular movements [11]. The three-dimensional analysis of the mandibular movements during mastication improves the understanding of the coordination of these complex functional movements. Thus it will be possible to observe patterns of three-dimensional movements in normally not considered planes and in the future to evaluate possible changes/deviations from these planes.

In addition, the individual analysis of each masticatory cycle represented a major breakthrough of this study, allowing orderly evaluation of the size, orientation, angle, and shape, from the first cycle to the last. No similar examinations were found in the literature obtained by this kind of analysis with the freedom that the 3D plot provides and particularly methods able to separate the trajectories of the set of chewing cycles. All the features related to cycle shapes reveal the complexity of this rhythmical motor-sensory activity [2–7]. The data suggest that the distributions of cycle orientation, shape, and prevailing side [10] are promising factors for assessment that can be evaluated after adjustments to the scripts used in these studies.

The individual cycles were evaluated from the frontal plane because it is the most relevant view in this case; however, the script used is sufficiently versatile to provide data cycles in different planes, this being another factor that could be improved in the development of this methodology.

Analysis of the number of masticatory cycles performed in the chewing process by subjects when no limits were set revealed wide variation ranging from 8 to 27 cycles recorded among the repetitions of five participants. The cycle area also showed a wide variation in both mean areas recorded among the subjects and the variance among cycles of the same repetition. These data show the variability in this type of movement between the different individuals evaluated and within the same “masticatory event.”

The activity and the number of masticatory cycles increase with the toughness of the food [7, 26, 27]. The number of 30 to 35 cycles is the approximate amount required before swallowing [26, 28]. The data obtained in this study with peanuts chewed by young subjects without articular and muscle problems suggests an average number of cycles (~21.5), 35–40% smaller than the values cited above, suggesting that this parameter can be modified by multiple factors. Van der Bilt in a review [7] reported that a large variation in the number of masticatory cycles performed for different types of food, specifically for the same volume of peanut, was registered to be 17 to 110 cycles before swallowing.

As mentioned above in discussion of the cycle areas, there was a wide variation in the values of this parameter from less than 4% up to 19% and high standard deviations in some cases equaling the mean value. These data reveal the wide variability of the cycles, especially related to the area they occupy in the frontal plane.

It was hoped that analysis of the distribution of the cycle area would reveal some kind of pattern in the distribution areas, for example, from larger initial cycles areas with the gradual reduction to the last cycles. The data in this parameter does not reveal any type of pattern that was repeated between the different subjects or between repetitions by the same subject. However it was noted that the number of cycles and amplitude of the areas in different repetitions by the same subject showed regular behavior.

Iguchi et al. [4] propose the division of mastication into three stages “initial, intermediate, and final.” This study only assessed the distribution of cycles and other features without divisions; a further customization of the scripts used may also include this division, allowing comparisons between phases.

The types of equipment currently used for recording mandibular movements are based on different principles such optoelectronics, electronics, and ultrasound [14].

Other devices employing the ultrasound principle “JMA (jaw-motion analyzer)” [11] and “ARCUSdigma, KaVo” [10, 29] and also the electromagnetic principles “K6-1” and “K7” Myotronics mandibular scanner and “Electrognathographer JT-3D” [8, 30–32] have been used to evaluate various clinical situations. These devices provide large amounts of data; however, independent of the principle applied, in all these cases, the presence of equipment parts, including facial arches fitted to the subjects' head and/or neck region, interferes with free, natural movement.

The AG501 EMA does not require any kind of immobilization; sensors are simply fitted in the regions of interest. Potential unwanted movements of the head and neck are excluded by the use of reference sensors attached to the head; these sets of sensors act as stabilizers of the masticatory movements; the movements recorded in the regions of glabella, mastoid, and upper incisors are automatically disregarded by the equipment and only the movements of the movement sensor located in the mandible are recorded, thus allowing movement to occur freely and naturally without need for physical stabilization.

Other studies use movement recording systems based on video systems [3, 33–35] which offer the advantages of low cost and greater freedom of movement since they do not require a facial arch or any other structure fitted to the subject's head. However, although these studies can generate large amounts of data, the data is difficult to interpret and the accuracy of measurements is impaired.

A system based on video recording, “Vicon MX 1.7.1,” was more user-friendly and smaller and with no arch or parts that immobilize the head and neck of the subject. It provides large amounts of data but does not record movement trajectories and can only record the displacement of the lower jaw in the vertical axis [36].

Analysis of mandibular movements by electromyography (EMG) is considered the gold standard for mandibular movement [33]. Many studies use this type of analysis, which delivers important data on muscle activity; however, while the activation/recruitment of muscles results in movement, the movement itself cannot be fully explained by this kind of isolated analysis. Thus many groups of researchers associate EMG with movement recording systems [6, 10, 11, 30] favoring broader understanding of mastication. However the systems described above for recording movement in this way produce simplified data or interfere with free movement, which could affect understanding of its complexity. Our research group intends to synchronize the records of movements obtained by the method presented here with EMG in the near future, further expanding our understanding of this complex function in different clinical conditions.

Among the limitations of this study, we should mention the small number of subjects, a homogeneous group without separation of data based on age, sex, and dental and skeletal classes, in which comparisons were not performed. Moreover, more features could be added that may be extracted from analysis of scripts, as mentioned in the discussion. However, it is normal for this type of limitation to appear during the development of such a new methodology. The adaptation/customization of scripts to obtain better analysis and a greater amount of scientific and clinical data is part of this development process. Finally, another limitation could be the presence of the cables that connect the sensors to the equipment, mainly due to the location of the motion sensor in the incisive mandibular region, in contact to the lips. The presence of these cables may influence the movements evaluated; however, it would not be possible to perform such analysis without the presence of these sensors and the option for wireless sensors would not be feasible, as they could cause interference in the equipment.

The results presented and discussed here demonstrate that the proposed method of masticatory cycle analysis achieved with the objective of providing reproducible, intelligible, innovative, and accurate quantitative and graphical data quickly and simply. It allows free, natural movement by the subject, free analysis from different levels and angles by creating 3D layouts, and individualization of cycle trajectories. These facts suggest that this method of analysis is promising, and may be applied in different studies evaluating different clinical situations and treatments. This method of analysis using the EMA-AG501 will continue its development by our research group, and in the sequence validation studies of this equipment for masticatory movements will be carried out.

## Figures and Tables

**Table 1:** Quantitative data of masticatory cycles.

	Number of cycles (mean)	Cycle area (mean ± SD, mm^2^)	FMBM area (larger, mm^2^)	Ca/FMBMa (mean ± SD, %)
Subject 1 (♀), 22 years	23	22.06 ± 7.66	452.88	4.870 ± 1.69
Subject 2 (♂), 18 years	13.67	74.67 ± 32.27	391.44	19.076 ± 8.24
Subject 3 (♀), 18 years	9	54.66 ± 34.1	436.29	11.564 ± 8.23
Subject 4 (♀), 18 years	21.5	21.49 ± 19.11	566.67	3.706 ± 3.38
Subject 5 (♂), 19 years	24	29.78 ± 17.6	421.97	7.057 ± 4.17
